# Goldilocks, vitamin D and sarcoidosis

**DOI:** 10.1186/ar4568

**Published:** 2014-05-23

**Authors:** Robert P Baughman, Elyse E Lower

**Affiliations:** 1 Department of Internal Medicine, University of Cincinnati Medical Center, Cincinnati, OH 45267, USA; 2 1001 Holmes Building, Eden Ave, Cincinnati, OH 45267-0565, USA

## Abstract

While low levels of vitamin D can increase the risk for osteoporosis, excessive amounts of vitamin D may also be problematic. Hypercalcemia and hypercalcuria due to increased vitamin D activity occur in a significant proportion of sarcoidosis patients. Saidenberg-Kermanac’h and colleagues compared vitamin D levels with bone fragility fractures in their sarcoidosis clinic. They found that a 25-(OH) vitamin D level between 10 and 20 ng/ml was associated with the lowest risk of bone fractures and paradoxically higher levels increased the risk of bone fractures. Using less vitamin D supplementation may simultaneously lower the risk for bone fracture and hypercalcemia in sarcoidosis.

## 

In the previous issue, Saidenberg-Kermanac’h and colleagues provide more information regarding the complexity of vitamin D activity in sarcoidosis [1]. A few years ago, vitamin D was declared the nutrient of the decade. This was heady stuff for a sterol that was originally felt important only in preventing rickets. Studies have demonstrated its key role in calcium absorption and bone growth. Beyond that, vitamin D has been considered an important sterol in various aspects of health. Low levels of vitamin D have been associated with increased risk for cancer, type 2 diabetes, and heart disease.

Most of these observations have been based on measurements of 25-(OH)-vitamin D3 (ergocalcitrol). This sterol is converted by 1-alpha hydroxylase to 1,25-(OH)2-vitamin D3 (calcitrol), the active form of vitamin D. This conversion occurs in the kidney and patients with chronic renal failure require calcitrol replacement.

In tuberculosis, vitamin D supplementation has been recommended in patients because vitamin D is crucial in the granulomatous reaction against the organism. However, what may be good for tuberculosis eradication may not be good for sarcoidosis. It has been noted that excessive amounts of vitamin D are associated with a worse clinical outcome in sarcoidosis [2]. In granulomas, there may be increased activity of 1-alpha hydroxylase. As part of the Th-1 immune response, calcitrol has a paracrine effect within the granuloma. In some cases, this leads to excessive calcitrol, resulting in hypercalcuria or hypercalcemia [3]. At least 10% of sarcoidosis patients have hypercalcemia, half of whom can develop associated renal dysfunction [3,4]. In some cases, hypercalcemic renal failure can be reversed by simply withdrawing vitamin D supplementation [3]. There are case reports of excessive vitamin D replacement leading to hypercalcemia in patients with mycobacterial infections [5].

The sarcoidosis patient may be treated with glucocorticoids, sometimes for years. Obviously, long-term glucocorticoid administration places the patient at risk for developing osteoporosis [6,7]. In rheumatoid arthritis, patients undergoing prolonged glucocorticoid treatment are recommended to receive calcium and vitamin D replacement [8]. While this is the cornerstone of prevention of osteoporosis, the role of calcium and vitamin D replacement in sarcoidosis remains unclear [9].

Into this quandary comes the study by Saidenberg-Kermanac’h and colleagues reported in the previous issue of *Arthritis Research & Therapy*[1]. After studying a large cohort of sarcoidosis patients from their clinic, the authors found that fragility fractures occurred in nearly a quarter of them. The fracture risk was increased for those treated with corticosteroids. Although low levels of ergocalcitrol was an independent risk for osteoporosis, ironically high levels of ergocalcitrol were also associated with an increased risk for osteoporosis. They found that ergocalcitrol levels of 10 to 20 ng/ml was associated with the lowest fracture risk for patients. This J shaped risk for bone fragility has been noted in non-sarcoidosis patients, although the proposed target levels are higher for these patients [10]. For the clinician treating sarcoidosis, one has to balance not only the risk for osteoporosis, but also the risk for hypercalcemia and renal failure (Figure 1).

**Figure 1:**
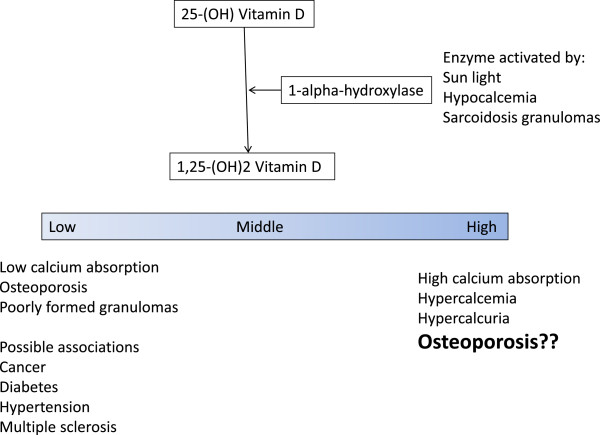
**Schematic depicting vitamin D metabolism in the body from ergocalcitrol to calcitrol**. The conversion is enhanced by increased activity of 1-alpha hydroxylase activity in the granuloma of sarcoidosis patients. The untoward consequences of low or high vitamin D activity are summarized at the bottom of the figure.

One possible explanation for the lower ideal ergocalcitrol level in sarcoidosis is the enhanced activity of 1-alpha hydroxylase in sarcoidosis granulomas. The authors did not provide information regarding calcitrol levels in their patients. The proportion of calcitrol to ergocalcitrol appears to be higher in sarcoidosis compared to non-sarcoidosis conditions. In one study of 270 sarcoidosis patients, 80% had low ergocalcitrol levels, but less than 1% had low calcitrol levels. In fact, that study found that 10% of patients had elevated calcitrol levels [3]. Those with elevated calcitrol were more likely to have a history of hypercalcemia or hypercalcuria. Higher levels of calcitrol have been associated with more advanced pulmonary sarcoidosis [2].

The other potential benefits of vitamin D replacement in sarcoidosis are unclear. Should sarcoidosis patients with low ergocalcitrol but normal calcitrol levels be prescribed vitamin D supplementation to reduce their risk for cancer and type 2 diabetes? If so, do they increase their risk for hypercalcemia or hypercalcuria? Could this increased vitamin D intake raise the functional level of vitamin D even higher and therefore increase the risk for osteoporosis?

To paraphrase Goldilocks, one does not want too little or too much vitamin D. You want just the right amount.
